# A Methodology for Reconstructing Source Properties of a Conical Piezoelectric Actuator Using Array-Based Methods

**DOI:** 10.1007/s10921-022-00853-6

**Published:** 2022-02-21

**Authors:** P. A. Selvadurai, R. Wu, P. Bianchi, Z. Niu, S. Michail, C. Madonna, S. Wiemer

**Affiliations:** 1 Swiss Seismological Service, ETH Zürich, Zürich, Switzerland; 2 Engineering Geology, Department of Earth Sciences, ETH Zürich, Zürich, Switzerland; 3 Structural Geology and Tectonics Group, Geological Institute, Department of Earth Sciences, ETH Zürich, Zürich, Switzerland; 4 Department of Earth Sciences, ETH Zürich, Zürich, Switzerland

**Keywords:** Piezoelectric transducers, Absolute calibration, Spectral deconvolution, Waveform modeling, Force–time function

## Abstract

We investigated the force produced by a conical piezoelectric (PZT, lead zirconate titanate) transducer actuated by high voltage pulses (HVP) in contact with a steel transfer plate. Using elastic wave propagation theory in a semi-infinite plate, we aimed to quantify the magnitude and estimate the shape of the force–time function via the body waves produced in the transfer plate using the displacement field recorded on an array of 20 absolutely calibrated PZT receivers. We first calibrated the receiver array using glass capillary fracture. We proceeded to use a conical PZT transducer to actively produce a source at the origin, allowing us to study the displacement field produced on the now calibrated PZT receiver array. We studied two types of HVP: An impulsive and step source. The calibrated receiver array was used to estimate the general shape of the force–time functions for each type of HVP. From our hypothesized force–time functions we were able to estimate the peak force produced by the PZT actuator: The impulsive source generated a force of $f_{peak} = 2.90 \pm 0.42$ N and the step source generated $f_{peak} = 1.79 \pm 0.30$ N, respectively, for a peak applied voltage of 273 V. This translates to an applied force of $\sim$ 0.011 N/V and 0.007 N/V for the impulse and step force–time functions, respectively, which is similar to estimates found in the literature for other conical transducers in contact with metallic transfer media. This measurement was verified directly by independent measurements of the peak force $f_{peak}$ using a dynamic force transducer. We found that our methodology correctly estimated the magnitude of the force but is limited to transducers with incident angles $\theta<$ 53$^{\circ }$. Beyond this angle, overestimates of the force were observed due to the lack of body wave energy produced by the source. These results allow us to quantitatively determine the forces produced by active PZT techniques using only the measurement of the displacement field captured on a calibrated conical PZT array. Quantitative understanding of active PZT sources additionally constrains the transfer functions approach, which is commonly used in the non-destructive testing of materials and in other fields, such as rock physics and laboratory seismology.

## Introduction

Our study focuses on understanding the transient forces produced by active piezoelectric (PZT) elements when excited by a high-voltage pulse (HVP) and in contact with a steel transfer plate. By understanding details of the source signal produced by a PZT actuator, specifically the shape and magnitude of the force–time function, we can develop a unique calibration methodology for an array of PZT receivers in continuously changing and inaccessible environments. Understanding the characteristics of the actuator will allow a better interpretation of the mechanics of micro-crack damage associated with acoustic emissions (AEs). The measuring of acoustic emissions is commonly referred to as acoustic emission techniques [1].

In laboratory fracturing experiments AEs are used to track the micro-cracking and damage in rocks and other materials [2, 3]. AEs have also been used in seismology [19, 22, 28, 29] and are believed to be scaled to earthquakes [e.g. 23, 24, 27, 38] that produce displacements with higher frequencies (from 20 kHz to 1 MHz); the earthquakes occur at source dimensions scaled from tens of microns to millimeters. Measurements of AEs in the laboratory are performed using PZT transducers that operate under the same principle as seismometers. Therefore, they also suffer from similar problems, such as site (coupling) effects, electronic errors and the influence of the internal components of the seismometer/PZT transducer. Improved calibration methods have been employed in experimental laboratory work [4, 6, 11, 23, 26, 32, 34, 37, 40] to give a better understanding for modeling of the source that produced the elastic waves.

While improvements in AE techniques have benefited from absolute calibration, these methods are typically used in a “passive” sense; i.e., where sources are quasi “produced” within the material as defects nucleate cracks. However, these methods may also benefit from “active” testing. During active testing, the PZT transducer acts as a source and produces a wave field that is measured by other passive receivers. Active methods can also benefit from increased knowledge of the instrument response since the receivers are typically characterized using the concepts of transfer function [13, ch. 5]. Better quantitative understanding of the source produced by PZT transducers could help with various applications, such as the inspection of large structures (dams and bridges or thick-walled pressure vessels) [13]. Micro-electro-mechanical transducers can also be embedded in structures using guided waves for various inspection goals, provided that there are expanded avenues for deployment [14]. Lamb waves excited by PZT actuators have been used to detect cracks for damage identification and quantification in metal and composite plate-like structures [8, 15, 35].

Determining the precise nature of the force–time function is important in various field applications and allows for improved understanding of the physical problem. Fields such as biomedicine use ultrasonic tests, which study elastic waves generated from PZT actuators into the fluid medium require a fundamental understanding of the force- or pressure-time produced by the transducer. Christensen [5] looked at the pressure source caused by applying a high-voltage pulse to an ultrasonic transducer with a piezoelectric element in contact with a fluid environment. It was found that the transient response of the pressure did not precisely duplicate the waveform of the voltage due to the resonant properties of the crystal. When excited by an impulsive high-voltage pulse, the crystal resonates in a sinusoidal manner at its fundamental frequency and damping occurred due to internal, external and transmitted losses. The kinematics of the problem can be explained using the equations of motions for an under-damped spring-mass-damper oscillator. This was also found on a sensor-solid interface by Breckenridge et al. [4].

The magnitude of the force produced by PZT actuators in contact with an aluminum transfer plate has been studied using direct measurements of Breckenridge et al. [4]. They used a capacitive force transducer and estimated the force–time function for a variety of different sources: a glass capillary fracture (applied in this study), a conical transducer (applied in this study), ball impact, spark source and high explosive source. Their estimates of the peak force from conical NIST PZT sensors [34] was 1.58 to 1.63 N for an applied step voltage of 100 V and in contact with an aluminum transfer plate. We aim to use these force estimates as a baseline to validate our results. Sause and Hamstad [37] developed a numerical approach that uses a coupled structural and piezoelectric formulation, in combination with electric circuit modeling, to model conical and disc PZT sensors and their response to test force function, emulating the rise time of a glass capillary fracture (ASTM E1106-12). In their model, they were able to predict the voltage produced in the complex electro-mechanical system that comprise the sensors and also validated this against the modelled displacements for specific wave types (longitudinal and transverse waves, rod waves, Rayleigh waves and Lamb waves) that are commonly investigated in materials science and non-destructive testing and evaluation.

The work presented here is not directed towards calibration but uses these techniques as the starting point to study the force-time function produced by a PZT actuator. Using calibrated array of conical PZT receivers, we take advantage of the transfer function concept to describe the sequence of events linking the source to the recorded signal. We measure the wave field using a calibrated array of conical PZT receivers and show that we are capable of back-calculating the peak normal force produced by the source in a quantitative manner. Our methodology helps to quantitatively constrain active acoustic emission techniques produced using conical PZT actuators, which offers several benefits but has some drawbacks that will be discussed.

## Background

**Fig. 1:**
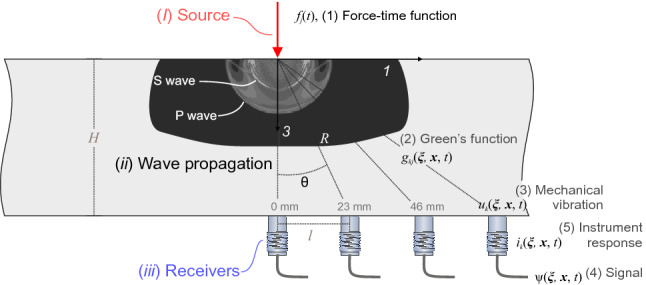
Lamb’s problem describing elastodynamic wave propagation in a plate due to a vertical surface point force (red arrow). The velocity field from a pulse-like source to illustrate wave phases in our transfer plate simply. The source is located on the top of a steel plate acting in the 3-direction and PZT receivers were mounted on the bottom of the transfer plate at varying off-epicenter distances *l* and incident angles $\theta$

The calibration of PZT transducers relies heavily on understanding the following: (*i*) the properties of the force–time function (source), (*ii*) the modeling of the wave propagation phenomena and (*iii*) the quantification of the instrument distortion imposed by the receivers. In Fig. 1, we show the five components of the “toolchain” that is used to link the source to the measured signal, which is governed by the equations of motion. The PZT actuator is used to produce (*1*) a **rapid transient point force** *f*(*t*) applied to the steel transfer plate at time *t*. Elastic stress waves propagate through the plate and the theoretical elastic displacement field throughout the plate can be solved using (*2*) **Green’s function** $g(\varvec{\xi },{\mathbf {x}},t)$. The Green’s function maps the force–time function to the (*3*) **mechanical vibrations** $u(\varvec{\xi },{\mathbf {x}},t)$ at point ${\mathbf {x}}$ and time *t* to the force at point $\varvec{\xi }$. A PZT receiver on the bottom surface at point ${\mathbf {x}}$ attempts to measure the theoretical displacements $u(\varvec{\xi },{\mathbf {x}},t)$. Inherently, the transducer will not be able to perfectly capture true displacements; each PZT transducer will uniquely distort the (*4*) measured signal $\psi (\varvec{\xi },{\mathbf {x}},t)$. This distortion can be quantified according to its (*5*) instrument response $i(\varvec{\xi },{\mathbf {x}},t)$, which includes the incident angle of the incoming elastic waves, the coupling effects between the transfer plate and senor face plate, the amplifiers and digitization effects and also the intrinsic features from the transducer construction. The incident angle is $\theta = \mathrm{tan}^{-1}(l/H)$ where *l* is the off-epicenter distance between the source–receiver in the ‘1’-direction and *H* is the thickness of the plate. The source–receiver distance is given by $R = \sqrt{l^{2} + H^{2}}$.

The general process for PZT transducer calibration is well-developed and was proposed decades ago. It focuses on the ability to produce known sources and the correct modeling of elastic wave propagation. Using a unique capacitive force transducer, artificial sources of AEs from pencil lead fracture, capillary fracture, conical transducer pulsing, ball impact, sparker sources, and high explosive sources have been investigated [4]. However, we aim to understand the source produced by a specific conical PZT actuator using only the displacement wavefield generated within the plate, employing Green’s function solutions for a steel transfer plate [e.g. 1].

We study waves in an elastic half-space, which was solved by Lamb [18] using the integral of plane wave solutions from Rayleigh [36]. We use a numerical code implemented by Hsu [16] to the problem outlined in Fig. 1, where we compute the mechanical disturbances from sources on an elastic half-space using the generalized ray theory approach.

We first focus on developing a broadband frequency-based understanding of the instrument response for an array of PZT receivers using well-documented capillary fractures [4, 23, 26, 38]. Once this was obtained, we developed a methodology to constrain the exact nature of the transient force–time functions produced when a rapid voltage transient is applied to a conical piezoelectric actuator.

## Theory

We revisit the theory surrounding Lamb’s problem to calibrate the PZT receiver array in the configuration described by Fig. 1. Due to spatial reciprocity of the representation theorem [1] and time-reversed solutions [10], we can use convolution of the system components to characterize our linear time-invariant (LTI) system. This approach assumes that changes in the transducers, source and material properties are unaffected over time. We assume point representations at both the receiver and at the source locations. Concepts related to PZT sensor calibration have been well documented and the treatment of the problem is well described [6, 21, 30, 31] and uses classical theory of elastodynamics [1].

We use the Green’s functions to relate the displacement field in a body at time *t* that is produced by a force at time $f_{j}$. Using elastodynamic theory, this can be done via the convolution of the force with the Green’s function and is shown below using the convolution integral,

1 $$\begin{aligned} \begin{aligned} u_{k}(\varvec{\xi }, {\mathbf {x}}, t )&= \int _{0}^{\infty } g_{kj} ( \varvec{\xi }, {\mathbf {x}}, t - \tau ) f_{j}( \tau ) \,d\tau \\&= g_{kj}( \varvec{\xi }, {\mathbf {x}}, t ) *f_{j}(t), \end{aligned} \end{aligned}$$

where the Green’s function, $g_{kj} (\varvec{\xi }, {\mathbf {x}}, t)$, is defined as the $k^{th}$ component of the displacement at ${\mathbf {x}}$ due to an impulse point force acting at $\varvec{\xi }$ in the $j^{th}$ direction initiated at *t* = 0. The force $f_{j}(t)$ is the point force component of arbitrary time dependence acting in the $j^{th}$ direction (summation over repeated indices is used). We note that $*$ represents convolution in time. Equation (1) represents the general case and in our analysis the force only acts in the 3-direction (see Fig. 1), i.e. $f_{j} = f \cdot \delta _{j3}$, where $\delta$ is the Kronecker delta. This simplifies (1) to

2 $$\begin{aligned} \begin{aligned} u_{k}(\varvec{\xi }, {\mathbf {x}}, t )&= g_{kj}( \varvec{\xi }, {\mathbf {x}}, t ) *f(t)\delta _{j3} \\&= g_{k3}( \varvec{\xi }, {\mathbf {x}}, t ) *f(t). \end{aligned} \end{aligned}$$

The voltage signal measured by the PZT transducers $\psi$ can be viewed using the convolution integral of the displacement field $u_{k}$ with the instrument response $i_{k}$, shown here:

3 $$\begin{aligned} \begin{aligned} \psi ( \varvec{\xi }, {\mathbf {x}}, t )&= \int _{0}^{\infty } i_{k} ( \varvec{\xi }, {\mathbf {x}}, t - \tau ) u_{k}( \varvec{\xi }, {\mathbf {x}}, \tau ) \,d\tau \\&= i_{k}( \varvec{\xi }, {\mathbf {x}}, t ) *u_{k}( \varvec{\xi }, {\mathbf {x}}, t ). \end{aligned} \end{aligned}$$

The instrument response depends on the position ${\mathbf {x}}$ (contact conditions, sensor and preamplifier, cables, digitization, etc.) but also position $\varvec{\xi }$ since the incident angle $\theta$ of the incoming waves affects the instrument response [40]. Similar to assumptions in the force, the recorded signals on the receivers are only measured in the 3-direction simplify the instrument response to $i_{k} = i\cdot \delta _{3k}$ and equation (3) to

4 $$\begin{aligned} \begin{aligned} \psi ( \varvec{\xi }, {\mathbf {x}}, t )&= i( \varvec{\xi }, {\mathbf {x}}, t )\delta _{3k} *u_{k}( \varvec{\xi }, {\mathbf {x}}, t )\\&= i( \varvec{\xi }, {\mathbf {x}}, t ) *u_{3}( \varvec{\xi }, {\mathbf {x}}, t ). \end{aligned} \end{aligned}$$

Combining (2) and (4), we can represent the measured voltage from the force that produced it,

5 $$\begin{aligned} \psi (\varvec{\xi },{\mathbf {x}}, t) = i(\varvec{\xi },{\mathbf {x}}, t) *g_{33}(\varvec{\xi }, {\mathbf {x}}, t) *f(t), \end{aligned}$$

for the problem described in Fig. 1.

**Fig. 2:**
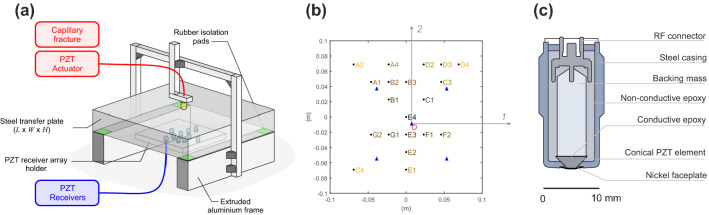
**a** General configuration of the calibration setup. The source was applied to the top surface of the steel base plate and an array of PZT receivers was attached to the bottom. **b** Displacements were measured using an array of 20 conical PZT receivers developed in-house. Additional tests were performed in a retrospective study to ensure repeatability of the methodology. The location of the five PZT receivers is shown with blue triangles. **c** Schematic cross-section of the PZT transducers used as both receiver and source

We use properties of the Fourier transform to interpret the system described in equation (5) in the frequency domain and take advantage of the fact that convolution and deconvolution are simply multiplication and division, respectively [3]. We can then rearrange equation (5) and solve for the instrument response *I* in the frequency domain:

6 $$\begin{aligned} I(\varvec{\xi }, {\mathbf {x}}, \omega ) = \frac{\varPsi (\varvec{\xi }, {\mathbf {x}}, \omega )}{ G_{33}(\varvec{\xi }, {\mathbf {x}}, \omega ) \cdot F(\omega )}, \end{aligned}$$

where *I*, $\varPsi$, *F* and $G_{33}$ are calculated from the temporal Fourier transforms of the *i*, $\psi$, *f* and $g_{33}$, respectively, and $\omega$ is the frequency.

By knowing the instrument response of the PZT receiver array, it is possible to study the spectral behavior of the unknown force–time function of the PZT actuator in contact with the steel transfer plate. To determine the instrument response given in Equation (6), we use capillary fracture as the calibration source at location $\varvec{\xi }$ at the origin *O*, which is discussed later.

### Quantifying the Force–Time Function of the PZT Actuator

Once the PZT receiver array is calibrated, the next step is to perform active pulsing of a PZT actuator at the collocated position (*O*) where the capillary tubes where fractured. This allows us to use the *a priori* knowledge of the unique instrument response calculated for our exact PZT source–receiver layout. The aim is to isolate properties of the force–time function produced by the PZT actuator in the frequency domain.

Next, by reconsidering the original problem in Fig. 1, we benefit from the known instrument response, which enables us to determine the displacement spectra from the measured signal. By rearranging Equation (6) we can isolate the force–time spectra:

7 $$\begin{aligned} \begin{aligned} F(\omega )&= \frac{\varPsi ( \varvec{\xi }, {\mathbf {x}}, \omega )}{I ({\mathbf {x}},\omega )\cdot G_{33}(\varvec{\xi }, {\mathbf {x}}, \omega )}. \end{aligned} \end{aligned}$$

In this study, we assumed properties of the force–time function. Firstly, the magnitude of force produced by a specific level of HVP generates a linearly proportional increase in the peak force. This assumption will be justified in the validation experiment in Sect. 6.5. This results in a force–time function in the time domain as follows:

8 $$\begin{aligned} f(t)&= f_{peak} \cdot f^{*}(t), \end{aligned}$$

where $f_{peak}$ is the force–time magnitude parameter and $f^{*}_{j}$ is the characteristic shape of the unit-force behavior of the force–time function. In the frequency domain we obtain:

9 $$\begin{aligned} F(\omega )&= f_{peak} \cdot F^{*}(\omega ). \end{aligned}$$

Our analysis will study $f_{peak}$ from the displacement measured on a calibrated array of PZT transducers in contact with a steel transfer plate.

## Experimental Methods

### General Facilities

Figure 2a gives a schematic representation of the steel plate (350 mm $\times$ 350 mm $\times$ 50 mm) in an extruded aluminium frame. Small rubber pads (30 mm $\times$ 30 mm $\times$ 3 mm) are used to isolate the vibrations in the steel plate from the frame [40]. The material properties of the steel plate that are required to model the wave propagation, are given in Table 1. The plate was assumed to be elastic, isotropic and homogeneous. An array of 20 PZT receivers are mounted in an aluminium array holder that is in contact with the bottom of the plate. Sources were applied to the center of the top surface of the plate since, in this study, we were interested in body waves.

**Table 1:** Properties of the steel (HABA CK45) transfer plate used in this study

Parameter	Symbol	Value
Density	$\rho$	7850 kg/m$^{3}$
Young’s modulus	*E*	210 GPa
Poisson’s ratio	$\nu$	0.27
P-wave velocity	$C_{p}$	5782 m/s
S-wave velocity	$C_{s}$	3245 m/s
Dimensions	*L*	350 mm
	*W*	350 mm
	*H*	50 mm

Two types of sources were applied to the same position on top of the steel plate: (1) a glass capillary fracture and (2) the dynamic actuation force from the PZT actuator. The glass capillary generated a well-constrained step-like force–time function [e.g. 4, Breckenridge1990], which was used to determine the instrument response for the individual source–receiver pairs of the array. Once the instrument response was calculated, we proceeded with our analysis of the displacements produced by pulsing the PZT actuator. A 273 V high-voltage pulse was applied to the PZT actuator using a pulsing unit (HVP, Elsys Instruments AE-HV-MUX) consisting of a Piezosystem Jena voltage amplifier for pulse generation (HVP 1000/200), which was fully integrated with the data acquisition system software (Elsys Instruments TraNET/Lab-AX).

Figure 2b shows the array holder for the 20 PZT receivers developed in Rock Physics and Mechanics Laboratory at ETH Zurich. In this study, we assumed that our source was symmetric about the *1-3* and *2-3* planes, since the source is aligned in the *3*-direction. The darker to lighter colors represent the increasing incident angles $\theta$ from the source, respectively. The receiver array was connected through a pre-amplifier system (Elsys AE-AMP) allowing us to choose either 0 dB, 20 dB or 40 dB gain settings. The pre-amplifiers impose a band pass filter with a -3 dB fall-off below and above the low and high cut frequencies, respectively. The low cut occurs at 5 kHz for all gain settings (0 dB, 20 dB and 40 dB) and the high cut varies between 5.0 MHz (0 dB), 3.0 MHz (20 dB) and 2.0 MHz (40 dB) [2].

A full suite of additional tests were performed in a retrospective study to (*i*) obtain estimates of the epicenter response ($\theta$ = 0$^{\circ }$) and (*ii*) ensure the methodology was repeatable. The blue triangles in Fig. 2b represent the locations of five additional PZT receivers used to verify the above points (*i*) and (*ii*).

### Design of the High-Fidelity PZT Transducers

Figure 2c shows the cross section of the custom-built PZT transducer, which follows the design of other point contact transducers [6, 7, 11, 34, 37]. The crystal in the sensor uses a 2.5 mm tall truncated cone of PZT-5a (a variant of the lead-zirconate-titanate ceramic). General properties of PZT-5A are given by Sause and Hamstad [37]. The tip of the cone (or aperture) has a 1.5 mm diameter with a base of 6.5 mm diameter, which is standard. The crystal geometry follows that detailed by the National Institute of Standards and Technology (NIST), which is widely used for wide-band conical PZT transducers [6, 12, 34]. This design minimizes the aperture effect by keeping the contact tip small, thus resolving the higher frequencies of the elastic waves. The conical shape also reduces the normal modes common to disc-shaped PZT elements due to its sensitivity to motion along the axial direction.

Previous research has shown that the backing mass must be carefully chosen; it is a critical component of a PZT transducer [11, 34]. The backing mass was fabricated from brass following the method of Glaser et al. [11] and was machined from a rod of 7 mm diameter with a height of 15 mm. Multiple cuts were taken on the back end of the mass with respect to the longitudinal axis, which reduced symmetry and prevented direct reflections from re-exciting the piezoelectric element [9, 34].

Fick and Proctor [9] described the electrical connection between the active element and the backing mass as a thin layer of low temperature (60$^{\circ }$ C) solder. This was not feasible for our construction as it needed to withstand temperatures in excess of 170$^{\circ }$ C in other applications. These constrains lead us to use a thin layer of a two-component silver conductive epoxy resin adhesive (Ag-epoxy, APS EP-01A/B) that had a resistance of 0.3$\times$10$^{-4}$ to 1$\times$10$^{-3}$ $\varOmega \cdot$cm for all the electrical connections in the transducer. The Ag-epoxy was used to assemble: The interface between the backing mass and PZT element, connecting the radio frequency (RF) pin (Coaxial PCB MCX-50-0-19/111-N-1) to the backing mass, connecting the thin nickel face plate (0.03 mm thick) to the tip of the PZT element and connecting the steel housing canister to construct the cathode of the system. The amount of Ag-epoxy used was reduced to minimize the gaps that completed the electrical circuit; this was found to reduce the noise floor of the transducer but small variations may influence the acoustic impedance, as discussed later.

A strong two component laminate resin (RenLam LY5210/HY5212) was used to encase the internal stem assembly consisting of the PZT element-backing mass-radio frequency (RF) connector. This embedded stem was inserted into the stainless steel outer casing (EN 1.4301) and secured with a press-fit backing ring and a small amount of silver epoxy. The outer casing was threaded (M14 x 1.25) used to mount the sensors to the array on the bottom of the steel transfer plate (Fig. 2a).

## Absolute Calibration of PZT Receiver Array

### Glass Capillary Fracture

The fracture of glass capillary tubes is a well-known mechanical source that initiates a high-frequency and wide-band response from the PZT receivers [e.g. 4]. The process relies on taking a length of thin-walled glass capillary tubing and loading it perpendicular to its longitudinal axis. We used a 0.1 mm (± 3%) outer diameter borosilicate thin-walled capillary tube (0.01 mm thick) with a side length $\sim$ 15 mm. The glass capillary is slowly loaded by turning a micrometer screw that loads in the direction perpendicular to the cylindrical axis of the capillary tube. An in-line dynamic force transducer (PCB-208C01) was used to measure the average force drop $f_{amp} \sim$ 17 N (± 4 N). The exact shape of the force–time function was not captured since the frequency resolution of the force transducer was limited to 10 kHz. However, the dynamic fracture of the capillary is assumed to have a rise time $t_{rise}<$ 200 ns as observed by Breckenridge et al. [4].

Using the capillary fracture, we computed each instrument response using the body wave for each source–receiver pair. A minimum of 9 capillary fractures were performed at each gain setting of the pre-amplifiers (0 dB, 20 dB and 40 dB). Our instrument response implicitly captured the effect from the sensor assembly itself, the contact mode at the transducer-plate interface, and the electrical effects (cabling, pre-amplifiers and digitization).

**Fig. 3:**
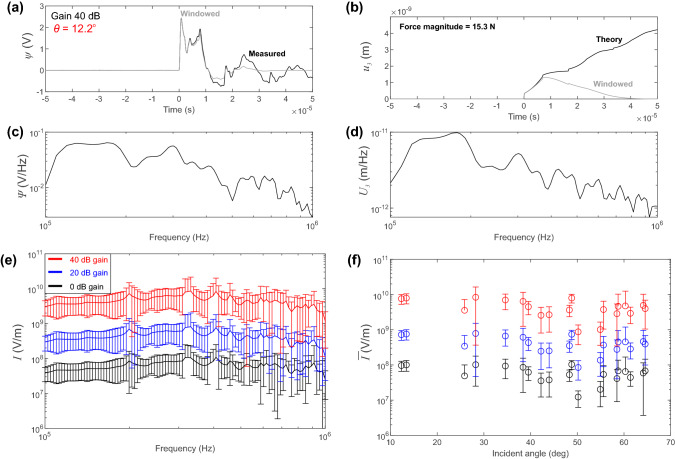
Calibration results from capillary fractures at point *O*. **a** Voltage response on sensor PCT-016 at $\theta$ = 12.2$^{\circ }$ at 40 dB gain settings on the pre-amplifier. **b** Theoretical displacements calculated at the co-located position determined using convolution of the force–time function and computed Green’s function. **c** Comparison of the spectral responses of the windowed voltage and **d** theoretical displacement. **e** Average instrument response between 100 kHz and 1 MHz for all receivers for different gain settings. **f** Average sensitivity of each receiver with respect to its incident angle $\theta$

### Calibration Results

Figure 3 shows the results for capillary fracture tests performed at point *O* on the top of the steel plate. Voltage signals $\psi (t)$ recorded by the PZT receiver are shown in Fig. 3a for a single capillary fracture at 40 dB gain. The receiver, chosen at random for demonstration purposes, is located at an incident angle $\theta$ = 12.2$^{\circ }$ and off-epicenter distance = 11.1 mm.

To calculate the Green’s functions $g_{kj}$ (Equation (1)), we adopt the generalized ray theory using the computational code given by Hsu [16] and adapted to MATLAB by McLaskey and Glaser [26]. This code has been validated with theoretical studies [e.g. 17, 33] and finite element models [25, 37, 39, 40]. Since we only considered the high-frequency behavior of the body waves induced in the plate (between 100 kHz to 1 MHz), we assumed that the plate is semi-infinite; reflections from the sides of the plate were ignored and only those from the top and bottom surfaces were modeled. The analysis time was restricted to < 50 $\mu$s [37], corresponding to the first reflected P wave to the outermost receiver in the array. Figure 3a shows a sample raw (black) and windowed (gray) waveform from receiver PCT-024 that was produced from a single glass capillary fracture at the origin. Theoretical displacements from the glass capillary fracture $u_{3}$ were computed using convolution in the time domain ($g_{33}*f_{3}$). Figure 3b shows the theoretical surface normal displacements $u_{3}$ calculated for the sample glass capillary fracture. We measured the force magnitude (= 15.3 N) obtained from the force transducer with an assumed rise time $t_{rise} =$ 200 ns to estimate the force–time function.

**Fig. 4:**
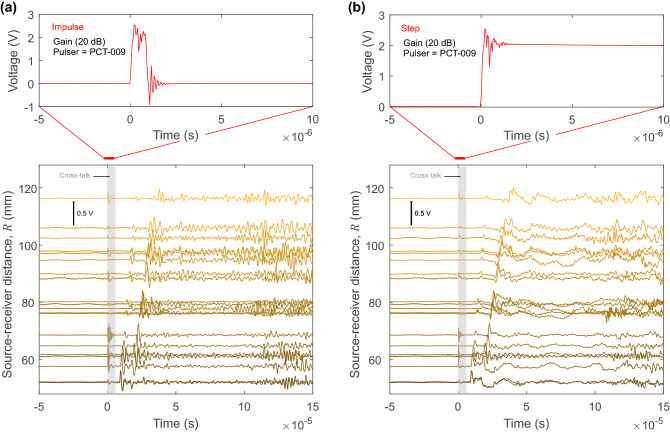
Results from the active PZT experiments. The top panels show the voltage measurement from the channel of the PZT actuator for the impulsive high-voltage pulse. The bottom panels show the distance versus travel-time plot with the array of 20 PZT receivers. Results are from the active PZT experiments with **a** impulsive or **b** step HVPs

Figure 3c and d show the spectral response for the experiment and theory, respectively. Spectral division (Equation (6)) was used to quantify the instrument response (*I*) for each receiver at its appropriate location. Figure 3e shows the average instrument response from 100 kHz to 1 MHz for all 20 PZT receivers in the array at the different gain settings. The average instrument response was calculated from 200 different measurements and the sensitivity ${\bar{I}}$ was interpreted as the average magnitude of instrument response across the bandwidth of interest. We found ${\bar{I}}$ = 6.3$\times$10$^{7}$ V/m (0 dB gain), ${\bar{I}}$ = 4.6$\times$10$^{8}$ V/m (20 dB gain) and ${\bar{I}}$ = 4.6$\times$10$^{9}$ V/m (40 dB gain). We see that the in-house developed PZT receivers used here perform as displacement transducers, due to the relatively flat-response with respect to the displacement spectrum between 100 kHz and 1 MHz, which is similar to the behavior of other conical shaped PZT variants described in the literature.

In the primary analysis, we employed the site specific instrument responses, but the purpose of examining the average receiver response in Fig. 3e was to confirm that the conical transducers do not have large resonance between the frequency bands 100 kHz to 1 MHz. As shown, the instrument response appears relatively flat, which is expected since the design of the PZT element-backing mass followed instructions from the literature [9, 11, 34]. In Fig. 3f we show the sensitivity ${\bar{I}}$ of each receiver with respect to the incident angle $\theta$. Incident angles varied from 12.2$^{o}$ to 64.6$^{o}$ over the array of receivers. The sensitivity was calculated as the average values of the instrument response from 100 kHz to 1 MHz at the given incident angle and the error bars indicate ± 1 standard deviation.

**Fig. 5:**
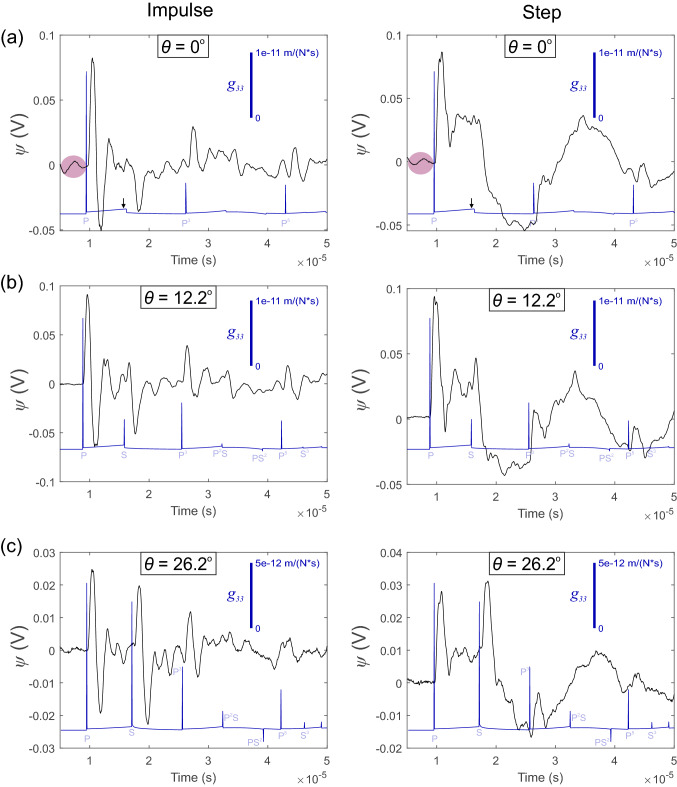
Example of the waveforms recorded on PZT receivers at **a**$\theta$ = 0$^{\circ }$, **b** $\theta$ = 12.2$^{\circ }$, and **c** $\theta$ = 26.2$^{\circ }$, for the impulsive (left panels) and step-like source (right panels). Signals were recorded at 20 dB gain. The waveforms are the result of stacking and averaging 50 traces (black line). Using the generalized ray theory [16], the true Green’s functions are given along with the theoretical wave arrivals including reflections (blue line). The purple regions in **a** emphasize the cross-talk from the HVP in the system at the lowest source–receiver distance

## Results from High-Voltage Pulsing Experiments

### Time Domain Analysis

Figure 4 shows the results of a HVP experiment using a PZT actuator (PCT-009) at the origin *O*. Although the HVP is applied, the exact shape of the transient voltage pulse is unknown. The signal recorded from the actuated sensor is digitized, which gives us some understanding of the applied high-voltage as shown in the top panels of Fig. 4. The HVP has two settings; the first is described as an impulsive source where the voltage is increased from 0 to 273 V and back to 0 V over one microsecond (top panel in Fig. 4a). The second source is a step-like source, as shown in the top panel in Fig. 4b. Here, the voltage is applied over one microsecond, held for 200 $\mu$s and then released (the release is not shown). For the waveforms analyzed in this study, this source was treated as step-like (the wave-field at 200 $\mu$s was not considered due to the reflected modes in the plate). We observed electrical interference (cross-talk) over most channels due to the HVP and we believe that this was likely produced on channels attached to the PZT actuator. The top panels in Fig. 4 were not representative of the actual voltage, but they were necessary for us to understand how the system was performing. In both cases, the pre-amplifier gain setting on the PZT receivers was set to 20 dB for this test.

We found that each source type produced distinctly different wave fields as determined by the travel-time distance plots made from the receiver array. The lower panels in Fig. 4 show the voltage traces recorded on the receiver array shown in Fig. 2b. Colors are related to the source–receiver distance *R* from the source, with dark to light representing close to far receiver locations, respectively, similar to the colors shown for the receiver locations in Fig. 2b.

Figure 5 shows the signals produced from the impulsive (left panels) and step (right panels) sources on sensors located at (a) at the epicenter ($\theta$ = 0$^{\circ }$), (b) incident angle $\theta$ = 12.2$^{\circ }$ and (c) incident angle $\theta$ = 26.2$^{\circ }$. A total of 50 HVPs was used to generate waves that were captured by the PZT receivers (colored traces) and the average of this stack was used to reduce the noise and is indicated by the black line in Fig. 5. We used the same generalized ray theory computational code [16] discussed in Sect. 5.2 to plot the theoretical Green’s functions at each source–receiver pair and to indicate the wave arrivals (blue line) of P waves (P) and S waves (S); the number of reflections for each wave within the semi-infinite space is given by the exponent. We show the true Green’s functions, which have units m/(N$\cdot$s), but they are shifted below the experimental waveforms for clarity.

### Characterizing the Shape of the Force–Time Function

**Fig. 6:**
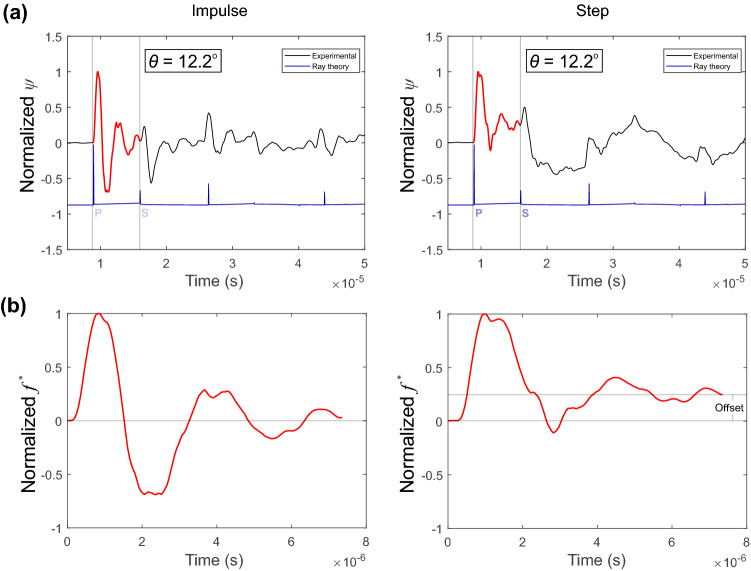
**a** Waveforms recorded at an incident angle $\theta$ = 12.2$^{\circ }$ for the impulsive (left panels) and step-like HVPs (right panels). Regions between the onset of the P and S waves are highlighted (red lines) for each source. **b** The isolated waveform in the top panel normalized by its maximum amplitude, which is deemed to represent the shape of our normalized impulsive (left panel) and step-like (right panel) force–time functions

To estimate the shape of the force–time function we rely on information gathered from the receiver at a relatively low incident angle $\theta$ = 12.2 $^{\circ }$. Figure 6a shows the averaged waveforms for 50 HVPs at this location for impulsive and step-like pulses. The waveforms between the P and S waves are highlighted in red, interpreted from the Green’s functions. Due to the convolutional nature of wave propagation, we assume that the voltage response has information related to the shape of the force–time functions. Our assumption is also strengthened by the sensor flat-response (Fig. 3). We choose to use the sensor located at $\theta$=12.2$^{\circ }$ to estimate the shape of the force–time function since there is a reduced near field effect from the S-wave that was present at the off-epicenter receivers at $\theta$=0$^{\circ }$ (see black arrows in the Green’s function in Fig. 5a).

Figure 6b shows the isolated waveform normalized by its maximum amplitude. We denote this as the unit force–time function $f^{*}$ and we see a distinct difference between the impulsive (left-hand side) and step-like (right-hand side) sources. The step-like source shows an amplitude offset from zero that was not present in the impulse source.

**Fig. 7:**
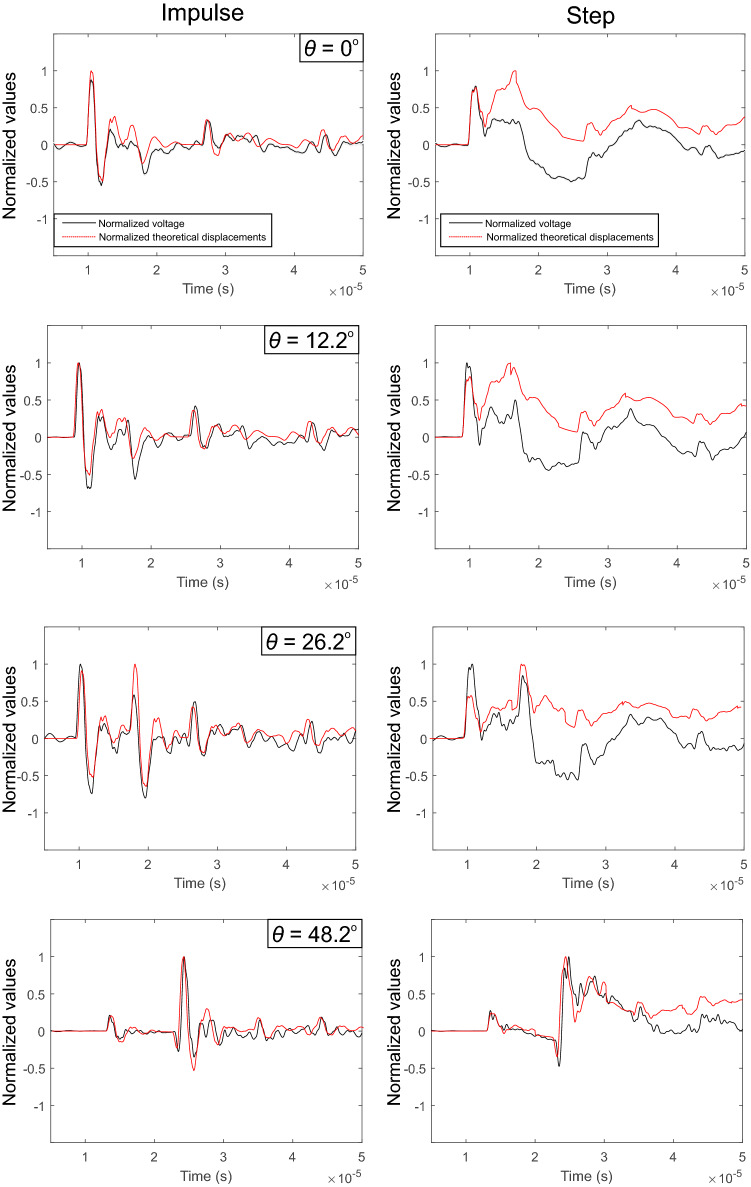
Examination of the validity of the chosen force–time functions (FT) in Fig. 6b for four sensors at increasing incident angles. Estimates of the normalized theoretical displacements are compared to the raw voltage measurements for the impulsive (left panels) and step-like (right panels) sources. Normalized theoretical displacements (red); normalized recorded waveform (black)

Since the choice of force–time functions needs to be verified, we tested our hypothesis qualitatively in the time domain. Using Equation (1), we convolved the force–time functions in Fig. 6b with the appropriate Green’s function. This operation gives the unit displacements for each normalized force–time function. Figure 7 shows the unit displacements (red) in comparison to the waveforms recorded (black) of four sensors at increasing incident angles. These results are given for qualitative purposes but the results meet the requirements to proceed with the quantitative analysis in the frequency domain that are presented next. Figure 7 shows normalized voltages and displacements for the impulsive (left panels) and step-like source (right panels) in the time domain. We see that the fits are qualitatively justified since the deviation observed to the step HVP is due to the offset in FT function shown in Fig. 6b.

### Frequency Domain Analysis

**Fig. 8:**
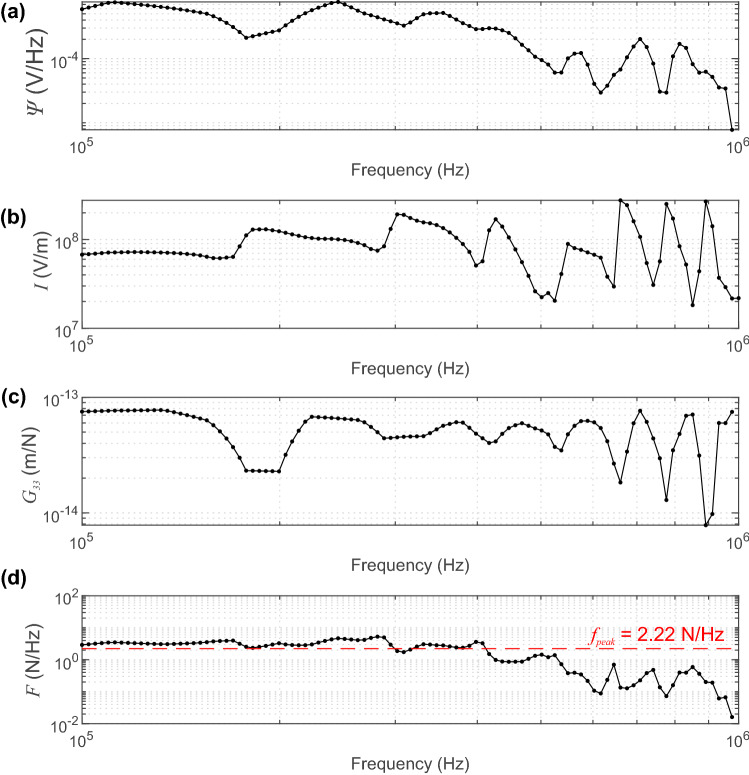
**a** Spectral response *S* for the waveform obtained at an incident angle of $\theta$ = 34.6$^{\circ }$ with 0 dB gain produced by a step HVP of $V_{step}$ = 273 V. **b** The instrument response *I* is calculated from the glass capillary tube fracture. **(c)** The theoretical Green’s function *G* of this PZT source–receiver pair. **(d)** The spectral representation of the force–time function is calculated using Equation (9). The estimate of the scaling factor (red dashed line) $f_{peak}$ = 2.22 N/Hz is the average spectral force between 100 kHz to 1 MHz

We apply the methodology described in Sect. 3 to estimate the scaling function (*SF*) determined through devconvolution in the frequency domain using Equation (9). To highlight the methodology, we show a single spectral analysis of the waveform obtained at an incident angle of $\theta$ = 34.6$^{\circ }$ with 0 dB gain produced by a step HVP of $V_{step}$ = 273 V at the origin. Figure 8a shows the spectral response of the waveform measured at the receiver ($\varPsi$). Figure 8b and c show the instrument response *I* determined from the glass capillary fracture and Green’s function $G_{33}$ between the source–receiver pair, respectively. Figure 8d uses these parameters to estimate the spectral force–time function *F* using Equation (9). The peak force ($f_{peak}$, red dashed line) is calculated to be $f_{peak}$ = 2.22 N/Hz and is the average spectral force between 100 kHz and 1 MHz in our analysis. The effectiveness of this average will be discussed later.

### Force–Time Function Scaling and Reconstruction

We used the average peak force $f_{peak}$ to correct the unit force–time functions for each PZT receiver, at each gain setting, and for both the impulsive and step sources. To do so, we estimated the general shape of the unit FT function in the time domain ($f^{*}$) using the assumptions made in Sect. 3. We then scaled the Fourier transform of the FT by the scaling factor ($f_{peak}\cdot F^{*}$).

**Fig. 9:**
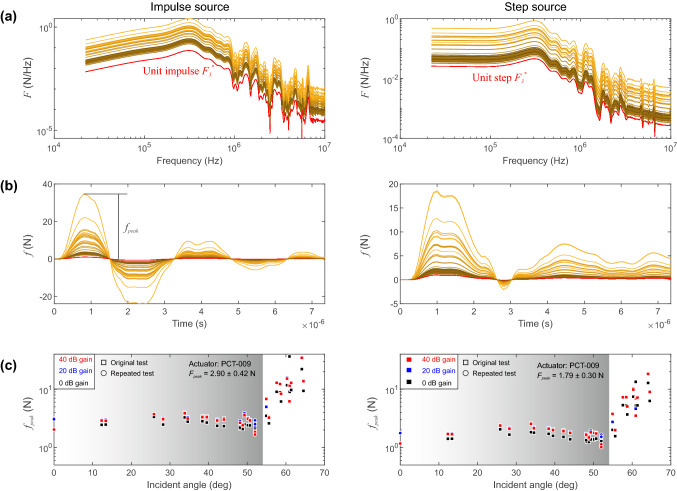
**a** Unit force–time spectra (red line) and corrected spectra (colored lines) for the impulsive (left panels) and step sources (right panels). Light and dark traces indicate higher and lower incident angles, respectively. **b** Reconstructed force–time function using the inverse Fourier transform [3]. **c** Comparison of the maximum force with respect to the incident angle ($\theta$). The test were repeated using five additional sensors (circle markers) and found that the methods employed are repeatable

Figure 9a shows the full spectrum of the unit force–time functions (red line) and the uniformly scaled force spectra (colored lines) for both the impulsive (left panels) and step source (right panels). The color is representative of the incident angle and off-epicenter distances from the source as given in Fig. 2b. We used uniform scaling *SF* over the full force–time spectra (gray shaded regions) to maintain the exact shape when returning to the time domain. The uniform scaling was motivated by the compelling qualitative results shown in Fig. 7.

Figure 9b shows the reconstructed force–time functions using the inverse Fourier transform. The unit impulse is shown in red and the scaled force–time functions are shown using the same color scheme as above. We observed systematic behavior when plotting peak force amplitude of the force–time function with the incident angle (Fig. 9c). Estimates of the force magnitude appear to deviate substantially at incident angles $\theta>$ 53$^{\circ }$ and this will be addressed in the discussion. We analysed the average peak force generated by the impulse and step sources of 273 V below the incident angle threshold ($\theta<$ 53$^{\circ }$) and for all gain settings. The average peak force $f_{peak}$ of the force–time functions from sensors was found to be $f_{peak}$ = 2.90 ± 0.42 N for the impulsive source, and $f_{peak}$ = 1.79 ± 0.30 N for the step source for our experimental configuration. For the applied voltage we calculated the force-voltage response to be $S_{L}$ = 0.011 N/V and 0.007 N/V for the impulsive and step sources, respectively. As mentioned in Sect. 4.1, to ensure repeatability of the methodology presented in this study, we performed an additional test with different array geometries and the results are given as circle markers. This required us to recalculate the instrument responses using identical capillary fracture techniques and the results are within reasonable limits, proving the repeatability of our methodology.

### Independent Estimates of Peak Force

While the force-voltage response $S_{L} \approx$ 0.009 N/V for the PZT actuator number PCT-009 was comparable to that found by Breckenridge et al. [4]($S_{L}$ = 0.01 N/V for an aluminum transfer plate), we performed tests to verify the validity of this estimate using an independent measurement of the force directly at the PZT actuator tip.

**Fig. 10:**
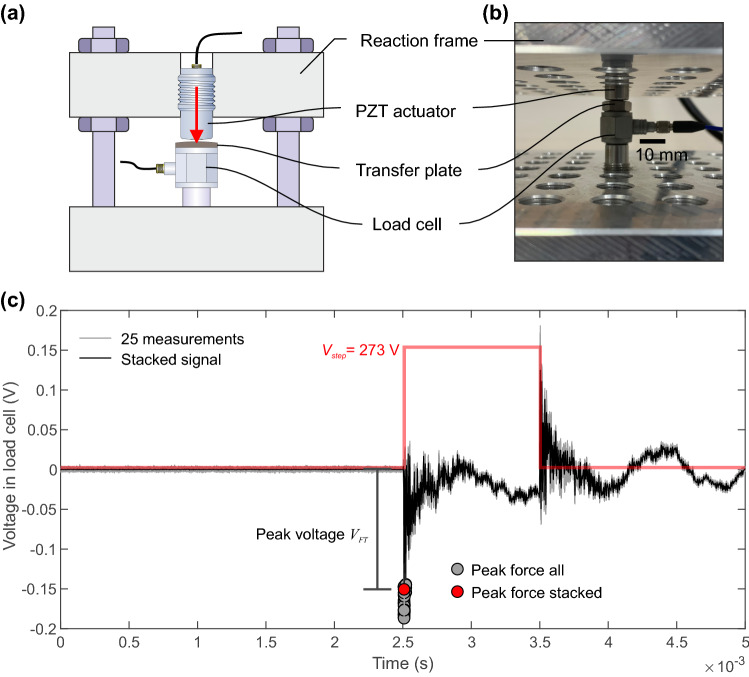
**a** A schematic depiction of the setup used to test the validity of the force produced from the PZT actuator and **b** a photo of the physical setup. **c** Voltage measurements in the dynamic force transducer for a applied step of $V_{step}$ = 273 V for 1 ms. All 25 measurement (gray) and the stacked response (black) is shown. The peak voltage was calculated for all measurements (gray circle) and the stacked signal (red circle)

Figure 10a gives a schematic depiction of the reaction frame used to test the validity of the force produced by pulsing the PZT actuator. A photograph of the physical setup is shown in Fig. 10b. The test consisted of loading the PZT actuator against a thin transfer plate that was coupled directly to the dynamic load cell (PCB-208C01). The load cell rested on a centralizing pin and the setup was pre-loaded before pulsing the PZT actuator. In these tests, only the step source was used since this could be compared to previous estimates of the peak force produced for an excitation step of 100 V of conical PZT actuators in contact with an aluminium transfer plate reported by Breckenridge et al. [4].

**Fig. 11:**
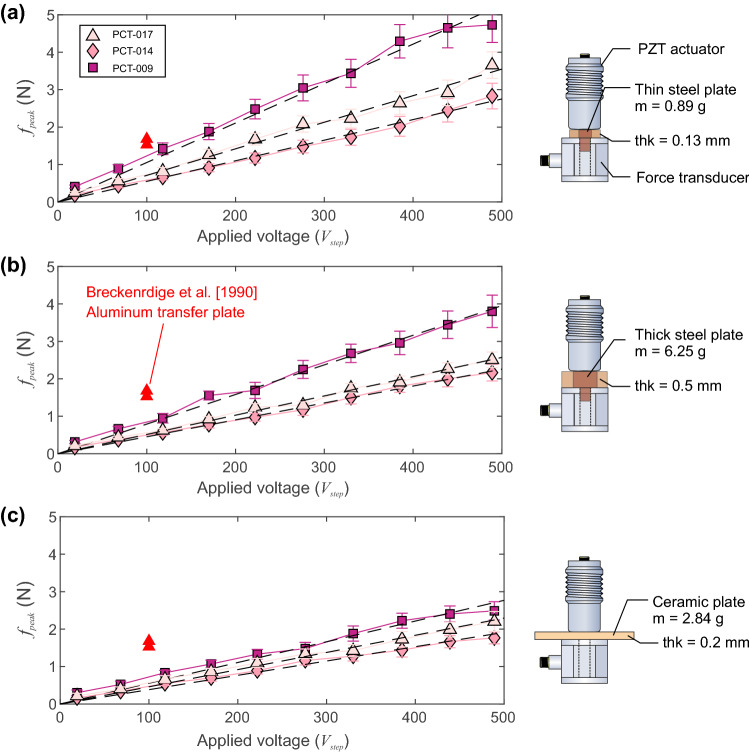
Results from the validation of the peak force using three different PZT actuators (PCT-009, PCT-014 and PCT-017). Three different load transfer plates were examined to see the effect of coupling: **a** thin steel transfer plate, **b** thick steel transfer plate and **c** a thin ceramic plate

Figure 10c shows a measurement made from the force transducer; pulsing the actuator “on” causes a negative voltage change due to compression. We take the minimum voltage reading recorded by the load cell since after the minimum peak was achieved, the force transducer dissipated the voltage and resonated before the voltage step was turned “off” 1 ms later. We assume that the lowest values measured in our tests are related to the peak force value described by [4, see fig. 11a therein]. A total of 25 pulses were performed at each step voltage excitation (gray lines) and the stacked response is also shown (black line). The minimum voltage value of each force measurement (compression is negative) is shown as gray circles and the stacked minimum voltage is given as the red circle. Figure 10c shows the waveform in the force transducer for a 273 V step over 1 ms. A total of 10 excitation voltages were studied with steps ranging from 36 to 489 V.

Results from the force validation tests are shown in Fig. 11. Three separate actuators were tested: PCT-009 (used so far), PCT-014 and PCT-017. We employed three separate loading transfer plates: a thin steel transfer plate with thickness 0.13 mm and weight 0.89 g (Fig. 11a), a thick steel transfer plate with thickness 0.5 mm and weight 6.25 g (Fig. 11b) and a thin ceramic transfer plate with thickness 0.2 mm and weight 2.84 g (Fig. 11c). Experiments using multiple transfer plates were initially performed since the force transducer had a threaded hole where the point of the PZT actuator would be in contact. However, by using three distinct loading plates we were able to check how the acoustic impedance of the transfer plate affected our understanding of the applied force at different levels of applied step voltage.

Three observations were made: (1) The peak force applied by the PZT actuator was linearly related to the applied step voltage for all three PZT actuators and for all three transfer plates. (2) Each sensor has a unique force-voltage response, which is likely due to small manufacturing differences related to the construction described in Sect. 4.2. The force-voltage sensitivity for the higher impedance thin steel transfer plate was $S_{L}$ = 0.0105 N/V (PCT-009), 0.0055 N/V (PCT-014) and 0.0071 N/V (PCT-017). For the lower impedance ceramic plate, $S_{L}$ = 0.0055 N/V (PCT-009), 0.004 N/V (PCT-014) and 0.005 N/V (PCT-017). Each PZT actuator performed in a predictable manner; the highest sensitivity actuator was consistently PCT-009 and lowest was PCT-014, irrespective of the transfer plate. (3) The impedance of the transfer plate played a role in the estimates of sensitivity for each PZT actuator.

For comparison, we included the single step voltage estimates from Breckenridge et al. [4] (Fig. 11, red triangles) who used a step voltage of 100 V applied to a similar conical crystal in contact with an aluminum transfer plate. We found that our estimates remained lower than those of Breckenridge et al. [4] with an aluminum transfer plate but were within a reasonable range, specifically when the applied force was estimated using the thin steel transfer plate (Fig. 11a) that may have had similar values of acoustic impedance to their study. These results confirm that our sensors behave in a linear manner and fall within the range of those reported by Breckenridge et al. [4] for another metallic transfer plate and typical NIST conical PZT designs [6].

### Validation of the Peak Force Using PZT Receivers

**Fig. 12:**
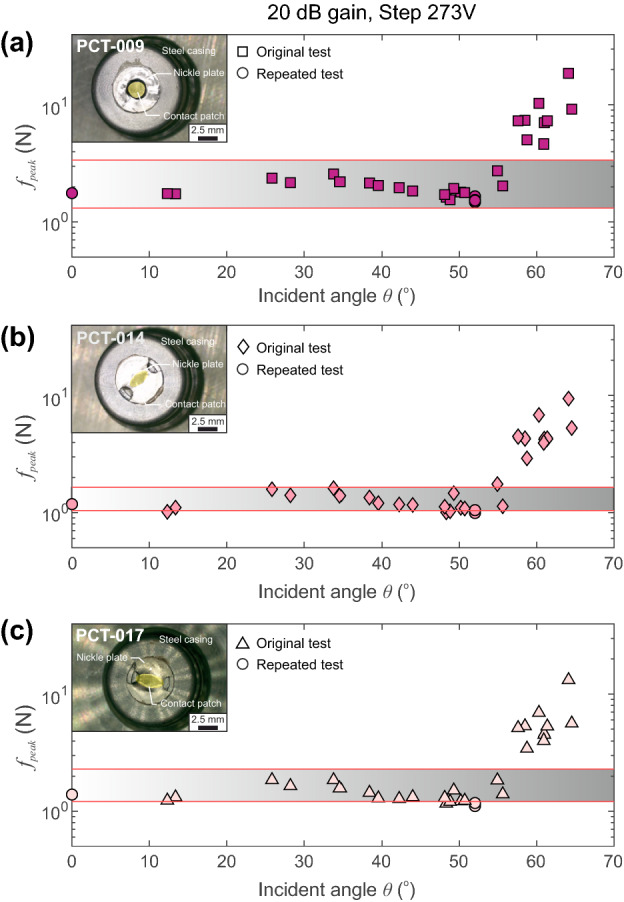
Estimates of the peak force from the deconvolution method of source reconstruction for a $V_{step}$ = 273 V for 20 dB gain on all three PZT actuators: **(a)** PCT-009, **(b)** PCT-014 and **(c)** PCT-017. The range of acceptable applied forces determined in the force calibration procedure in Sect. 6.5 is shown by the gray bounding box and the applied force were uniquely determined for each PZT source. We observe that the peak force estimates are well constrained at incident angles $\theta<$ 53$^{\circ }$

To check the repeatability of the peak force $f_{peak}$ estimated using the array-based method presented in Fig. 9, we deployed two more active PZT sensors that were tested directly in the previous section. Figure 12 shows the estimates of the peak force $f_{peak}$ for three PZT actuators: (a) PCT-009, (b) PCT-014 and (c) PCT-017. Results shown here were taken at 20 dB gain and estimated from the step source of $V_{peak}$ = 273 V. The results show that the general amplitude of the produced force falls within a range of force estimates given by the direct measurements up to an incident angle below $\theta<$ 53$^{\circ }$. Below this threshold, estimates of the peak forces using the PZT receiver array fall within the validated limits calculated in Sect. 6.5 and is represented by the shaded region in Fig. 12 (which differs slightly for each sensor). Estimating the average peak force at 20 dB gain for incident angles $\theta<$ 55$^{\circ }$ we found $f_{peak} = 3.2 \pm 0.4$ N (PCT-009), $f_{peak} = 2.08 \pm 0.27$ N (PCT-014) and $f_{peak} = 2.37 \pm 0.35$ N (PCT-017). The variations between the force estimates in each actuator were correlated to the variations in sensitivity made using the direct measurement approach. An explanation for this difference is discussed later. The inset images in Fig. 12 show detailed photographs of the nickel contact tip of the PZT actuators, which displayed (even visually) small variations in their construction.

## Discussion

The study of the transient point force $f_{j}$ in Equation (1) has been of interest to fields that employ AE techniques, such as laboratory seismology, rock physics, structural health monitoring and medicine. By quantitatively constraining the active PZT source using the array-based technique described here, allows for a more accurate understanding of the transfer function elements described in Fig. 1. In Sect. 5 we use absolute calibration methods to compute the instrument responses of an array of 25 PZT receivers by conducting glass capillary fracture experiments performed at the origin. Section 6 uses the now calibrated array of PZT receivers to estimate attributes of the force–time function produced by a PZT transducer, actuated at the origin, using only the elastodynamic stress waves. The peak force estimated from the PZT receiver array was then validated using independent measurements in Sect. 6.5. The aim of this study is to highlight the methodology, which has been shown to be effective for studying the properties of the transient point force $f_{j}$ produced by PZT actuators using array-based PZT receivers. While the results shown in Fig. 12 show this methodology is efficient, we briefly discuss some aspects that may contribute to variations in the overall estimates of the peak force and its sensitivity to source–receiver estimates at higher incident angles.

### Comparison to Peak Force Estimates

Using the theoretical concepts described in Sect. 3, we were able to reconstruct the peak force produced by the conical PZT actuators. It should be noted that intricacies in both the time and frequency domain are likely attributable to the various mechanical resonances of the conical transducer element and backing mass [4]. A review of the direct measurements on other conical transducers with identical PZT element geometry and material but differing backing masses, showed that the NIST conical variants [6] where found to deliver about $S_{L} \sim$ 0.01 N/V [4] with an aluminum transfer plate. We have made similar estimates here, using the reconstructed peak force from the calibrated receiver array for three different actuators: $S_{L}$ = 0.0105 N/V (PCT-009), $S_{L}$ = 0.0055 N/V (PCT-014) and $S_{L}$ = 0.0071 N/V (PCT-017). However, due to these discrepancies in force estimates between individual PZT actuators, we conducted separate experiments to directly measure the peak force, as explained in Sect. 6.5.

We found that the peak force versus applied voltage in our PZT actuators behaved in a predictable manner, showing a linear relationship between peak force and applied voltage on each sensor. The transducers also displayed variable sensitivity (Fig. 11) where PCT-009 to PCT-017 to PCT-014 showed decreasing levels of sensitivity. This sensitivity was also dependent on the acoustic impedance of the transfer plate and showed a predictable behavior when it was varied. This lead us to believe that the transducer construction, specifically at the tip, may influence the acoustic impedance of the nickle face plate/Ag Epoxy/PZT element composite structure that was slightly different in each transducer as shown in the inset photos in Fig. 12. While the tip construction of our conical sensors likely explains the variations in impedance and thus transmission of energy, these were also captured in the reconstruction of the peak force using the PZT receiver array. Over all gain settings, the less sensitive PZT actuator (PCT-014) produced lower force $f_{peak} = 1.93 \pm 0.34$ whereas the more sensitive sensor PCT-009 produced $f_{peak}= 2.96 \pm 0.47$ N. Both these results follow the trend and range of predicted peak force measured and were validated using direct force measurements in Sect. 6.5.

### Discrepancies in $f_{peak}$ Estimates at Higher Incident Angles

As we see in Fig. 12, estimates of the peak force begin to overestimate the predicted force range defined by the gray bounding regions at incident angles $\theta>$ 53$^{\circ }$. This error is systematic and occurs for all PZT actuators tested in this study. The systematic bias leads us to believe that this is not imposed by the mechanical and electrical components that showed variations of acoustic impedance due to discrepancies in the tip construction as discussed previously. We believe that it is due to the manner in which the *SF* is determined; simply as the average of $F(\omega )$ over the frequencies between 100 kHz and 1 MHz (Fig. 8(d)).

The incident angle increases past $\sim$ 53$^{\circ }$ accompanying an increased source–receiver ray path. In both tests, glass capillary fracture and high-voltage pulsing, the resulting normal displacement becomes relatively weak due to a decreased normal component (< cos(53$^{\circ }$)) and enhanced geometrical spreading. Therefore, the recorded voltage signal is also lower in magnitude in comparison to lower incident angles. This could introduce more error or uncertainty when calculating the maximum applied force using the averaging technique for $f_{peak}$.

We observed a similar deviation in the previous calibration work using the ball drop [40, see Fig. 9b therein]. Wu et al. [40] performed a series of ball drop tests with diameters ranging from 0.3 to 3 mm on the identical transfer plate and configuration used in this study. They derived the frequency upper limit, $\omega ^{max}_{\chi }$ corresponding to each diameter. $\omega ^{max}_{\chi }$ ranging from 100 kHz and 1 MHz is found in the case of ball diameter below 2 mm; however, they were not able to accurately fit with their ball impact source model when the incident angle was $\theta>$ 53$^{\circ }$. This observation is caused by a weak voltage signal at higher incident angles and is consistent with the findings of the peak force versus incident angle found here.

Another possible explanation for this overestimate is a larger source–receiver distance: more energy (e.g. anelastic attenuation or scattering) is dissipated as elastic waves propagate through the steel plate. This is an unlikely but valid hypothesis since the attenuation in steel at the frequencies looked at here presumed to be low [26]. Moreover, this dissipation is not considered in the current work.

Averaging of the scaling factor $f_{peak}$ from $F(\omega )$ and assuming the shape of the normalized force–time function $F^{*}(\omega )$ (see Fig. 9) may be an oversimplification of the problem; thus, we would not recommend using the current methodology to reconstruct peak forces produced by actuating conical PZT transducers with angles $\theta>$ 53$^{\circ }$. A possible improvement to this method may take into account frequency-dependent scaling factors but this is beyond the scope of this work.

## Conclusions

We followed the methodology for PZT transducer calibration that takes advantage of a theoretical understanding of the waves produced in a steel transfer plate from known force–time functions. We first calibrated an array of 25 PZT receivers, coupled to the opposite side of the transfer plate, using the capillary fracture method. Construction of our in-house transducers is detailed here; they show reasonably flat instrument responses between the frequency bands of 100 kHz and 1 MHz – a feature common to conical-type PZT transducers. Using the known instrument responses, the capillary source was switched for an active conical PZT source, that was actuated using impulsive and step HVPs. The differences between impulsive and step sources and the resultant displacement field was studied on the uniquely calibrated source–receiver array. Using spectral deconvolution, we were able to reconstruct the predicted maximum force produced from the PZT source via the measured waveforms through a steel transfer plate. We found that for the impulsive source the $f_{peak} = 2.90 \pm 0.42$ N while the step source generated $f_{peak} = 1.79 \pm 0.30$ N, both at peak applied voltages of 273 V. This translates to an applied force of $\sim$ 0.011 N/V and 0.007 N/V for the impulse and step force–time functions, respectively. We confirmed these estimates using a separate experiment to directly measure the peak force. If our methodology is followed, estimates of the peak force produced by pulsing a conical PZT actuator can be determined using only the wave field produced in the propagation medium, as measured by an array of conical PZT receivers.
